# Surgical Management of Impacted Lower Second Molars: A Comprehensive Review

**DOI:** 10.1055/s-0041-1739443

**Published:** 2022-01-11

**Authors:** Diane Isabel Selvido, Nattharin Wongsirichat, Pratanporn Arirachakaran, Dinesh Rokaya, Natthamet Wongsirichat

**Affiliations:** 1 Clinical Science Department, International College of Dentistry, Walailak University, Bangkok, Thailand; 2 Department of Preventive Dentistry, Division of Orthodontics, Faculty of Dentistry, Khon Kaen University, Khon Kaen, Thailand

**Keywords:** impacted lower second molars, molar impaction, lower third molars, surgical uprighting, surgery, extraction, impaction, second molars

## Abstract

Impacted lower second molars (ILM2) are rarely reported in the literature, but various studies have been done for its treatment. Apart from solely orthodontic approaches, different surgical management techniques were reported to have successful outcomes. Surgical intervention of ILM2 can help expose the tooth for further orthodontic purposes, simplifying complex treatment methods, and reducing treatment time. This review illustrates the comprehensive evaluation and updated methods of surgical uprighting, repositioning, and transplantation of ILM2 with future directions for better understanding and treatment planning in the clinical setting. The successful outcome of surgical intervention depends on case selection, root development of ILM2, careful surgical manipulation, and adherence to sound biological principles.

## Introduction

The nature of impaction of second molars has been observed to be an outcome of inadequate skeletal development to permit normal and undisturbed eruption. 1 2 The management of this occurrence has been challenging for orthodontists and oral and maxillofacial surgeons because of the technique-sensitive treatment planning, varying prognosis, and limited access to the tooth. 3 4 Objectives of imposing the tooth to be placed and positioned in its ideal position in the dentition are to avoid dental caries and possible inflammation of the periodontium in proximity to the distal of the first molar to inhibit arch discrepancies that may lead to instabilities. 3 Additionally, mastication muscles can be functionally affected by impacted second molars. 5

Second molar impaction is rarely reported in the literature, mainly because of its sporadic occurrence. 2 Together with first molars, it has a prevalence of 0.01 to 0.8%. 6 7 Moreover, among patients undergoing orthodontic treatment, the prevalence of this impaction was reported to range between 2 and 3%. 2 8 This type of impaction is frequent in the mandible compared with the maxilla and is described as unilaterally occurring in the dental arch. In addition, second molar impaction leans toward predilection in males compared with females, and is usually mesially inclined. 8 9 10

Management of impacted second molars has been discussed in the literature to be employing surgical or nonsurgical approaches. 5 As per Boynton and Lieblich, there is a consensus that conventional nonsurgical treatment, even if noninvasive, requires a long treatment period to complete. In addition, considerations like patient's adherence to proper oral hygiene and protocols and consistent orthodontic visits, especially in severe cases, are emphasized in the nonsurgical approach. 11 The surgical approach can be an optimum solution to some of the clinical dilemmas being faced by nonsurgical methodologies. 12 According to Anderson et al, surgical manipulation of impacted molars helps access the impacted tooth to bond with orthodontic brackets or buttons, assists in simplifying complicated treatment methods, and aids in reduction of treatment timelines. 3 The specific management varies from case to case and demands special attention since an impacted mandibular second molar can cause caries, periodontal problems, and resorption of the roots of the distal root of the first molar. 13 14 15 Several authors agree that surgery is an effective strategy in accessing, uprighting, and positioning impacted second molars. 5 16 17 These methods are deemed necessary since the result of manipulating impacted second molars, in general, can be unpredictable, and depends on timely detection and treatment. 7

The literature on ILM2 can be divided into two entities: surgical and nonsurgical. Although numerous case studies and retrospective studies are available about the surgical aspect, there has not been a review done exclusively about the various surgical techniques. In line with that, this review aims to illustrate a comprehensive account of the surgical management of ILM2 used in clinical settings.

## Methods

Electronic databases were utilized, such as Scopus, PubMed, PubMed Central, and EBSCO using associated keywords. The computerized search comprises case reports, literature reviews, clinical trials, retrospective studies related to mandibular or lower second molar impaction management, and surgical treatment modalities. All related topics searched were “surgical management” plus keywords such as mandibular or lower second impacted molars, surgical impaction managements, surgical uprighting, surgical repositioning, transplantation, autotransplantation, and other related subjects about lower second molar impactions. Publications in the English language were collected. Most of the literatures gathered in the indices were peer-reviewed articles related to impacted mandibular second molars, with 18 case reports and 6 retrospective studies.

### Initial Assessments

Similar to nonsurgical methods, a panoramic radiograph has been the imaging of choice of many authors in their respective reports. Panoramic radiographic imaging should be in conjunction with clinical examination to accurately identify the occurrence of impacted second molars and for postoperative assessment, especially when surgical intervention is considered necessary. Retrospective studies have relied upon these panoramic radiographs in ILM2. 14 18 Some studies also used periapical radiographs for a more detailed viewpoint. 19 20

According to Kravitz et al, a panoramic image can predict an upcoming ILM2 in a preadolescent patient when the third molar tooth follicles are placed on top of the incomplete second molar. 4 In surgical uprighting or repositioning and transplantation, numerous authors have synonymously noted the ideal age of treatment to be between 11 and 15 years old, with one-half to two-thirds of root formation. These parameters are best identified by panoramic imaging. 2 4 10 12

Another assessment being used today is cone beam computerized tomography [CBCT] which can identify obstructions in the eruption pathway. This particular evaluation was helpful in a report presented by Lorente et al 21 in a maxillary impacted second molar. CBCT can easily distinguish molar infraocclusions, molar angulations, and anomalies such as dilacerations that can deter the eruption of second impacted molars. Particularly, in the case of the ILM2, it serves as a valuable tool. 22 Since this method can identify any possible obstructions preventing eruptions of the said teeth in many planes of view, its utilization can assist significantly in maxillary and mandibular second molar impaction cases.

### Surgical Exposure of the Impacted Lower Second Molars

A careful assessment for surgical exposure of the ILM2 includes clinical and radiographic means. If the clinician observes that the ILM2 are not erupting within 6 to 12 months, a radiographic assessment can be performed to validate if there is an aberration or hindrance in the physiologic eruption pattern. 20 23 24 On the condition that the tooth is situated deep in its position, only surgical exposure may be indicated, and placing bonding attachments is unfeasible because of its difficult isolation. 25

Exposure is usually the first step in every surgical approach. It can be combined with other methods of uprighting second molars, including surgical luxation, surgical uprighting with miniscrews/miniplates, and orthodontic-assisted uprighting. 14

Kenrad et al 23 reported that surgical exposure for ILM2 had solved the problem regarding crown follicle retention, which causes the impacted second molar to not erupt at the expected range of time. They also stated that surgical exposure had the highest success rate of 90% among all surgical methods performed as spontaneous eruption was achieved. According to Magnusson and Kjellberg, 26 surgical exposure is the most effective treatment for impacted or retained second mandibular molars, with a 71% success rate for maxillary and mandibular second molars, particularly gaining positive results with a total of seven impacted second mandibular molars. Both maxillary and mandibular impacted second molars were discussed in their study, but the latter was used in the majority of the surgical exposure group that demonstrated favorable outcomes compared with other surgical approaches of impacted or retained impactions. 26 In a recent study by Abate et al, 27 operculectomy, a procedure where the clinician eliminates the soft tissue covering of an impacted or partially erupted tooth, which also resembles surgical exposure, successfully stimulated physiologic eruption when performed on 30 counts of ILM2. Their study also advised that vertically positioned and mesioangularly inclined second impacted molars benefit more from the case selection of this procedure.

The number of ILM2 in the studies mentioned earlier is very few, so further studies are needed. Nevertheless, overall outcomes have shown that surgical exposure is the easiest management that can be combined with other methods of surgical uprighting because of the simplicity of the procedure. A summary of retrospective studies regarding second mandibular molars utilizing surgical exposure is presented in Table 1 .

**Table 1:** Second mandibular molars utilizing surgical exposure on retrospective studies

Author	Age group	No. of second impacted mandibular molars	Outcomes
Kenrad et al 23	11 years 2 months to 19 years 8 months	10	Spontaneous eruption achieved
Magnusson and Kjellberg 26	1–19 years (mean age 15 years)	7 + 1 primary retained tooth	Most successful treatment outcome, 71%
Abate et al 27	Mean age 14.8 ± 1.3 years	30	Successful outcomes of 90.9% by spontaneous eruption

Second molar impaction is a rare occurrence, as previously mentioned. Thus, there is a lack of available studies about ILM2 in the literature worldwide. However, there are a few studies supporting that surgical exposure is the simplest modality to accommodate ILM2 eruption. Studies suggest that surgical exposure is more convenient and easier with orthodontic correction. Sawicka et al 28 reported that bilateral ILM2s were uprighted after surgical exposure and orthodontic uprighting. A study by Manosudprasit et al 25 also benefited from this method. Surgical exposure studies are shown in Table 2 .

**Table 2:** Case reports on surgical exposure of second mandibular molars

Author	Year	Second molars	Age, gender	Time of uprighting	Adjacent third molar status	Remarks
Sawicka et al 28	2007	37, 47	14 years old, female	5 months	Third molar was removed after uprighting	Orthodontically assisted
Manosudprasit et al 25	2013	37, 47	17 years old, female	13 months	Third molar was removed before uprighting	Orthodontically assisted

Wound healing problems would be a limitation of this procedure as impaired wound healing would cause further infection of the exposed site and incompliance. 29 Oral hygiene should also be reinforced for optimal healing of the exposed site.

### Surgical Positioning/Uprighting by Luxation

The decision to perform surgical uprighting should be made when all the conservative options for treatment are deemed to be unsuitable. 30 Many clinical implications are expected when surgical uprighting by luxation is involved, but it is still preferred over orthodontic extrusion since such a procedure was declared to have disadvantages. Surgical uprighting may prevent abnormality in the occlusal plane from the impacted tooth loss, supraeruption of the opposing tooth, and reduces the possibility of periodontal and prosthodontic problems in the future. 31 The results of this procedure were known to have a favorable prognosis, particularly when incomplete root formation is present. 4 18

Mindful surgical manipulation with minimal likelihood of injury to the cementum and periodontal ligament is advantageous in efficient bone regeneration. Following the concept of primary closure, osteogenic activity can be initiated, possibly developing mature bone on the crestal region. Thus, the importance of atraumatic and conservative uprighting and repositioning should be emphasized. 32

The process of luxating to an ideal uprighted position also has clinical restrictions when it comes to angulation. Pogrel indicated that the second molar should be slowly uprighted not beyond 90 degrees as this can lead to detrimental pulpal status postoperatively. 35 Cho et al suggested that the inclination angle between the first and second molars should be 75 degrees. 33 Both studies are congruent to each other as they indicate that exertion to angular limits would instigate behavior similar to uncontrolled transplantation that will negatively affect the tooth.

Peskin and Graber 34 advised the use of gelfoam, an absorbable gelatin sponge, placed around the prepared area in the event that the tooth is mobile after the operation. Similarly, in a retrospective cohort study by Caminiti et al, 14 they positioned a wedging sheet of surgicel to the mesial space to act as a stabilizer and a hemostatic agent on the luxated lower second molars. Another suggestion was from Dessner, where he instructed that the bone removed from the distal of the second molar prior to uprighting may be used as a wedge to the first molar to stabilize it. 10 An autogenous bone has also been mentioned to fill areas devoid of bone for splinting purposes. 5 32 However, Boynton and Lieblich 11 advocated that autogenous bone, bone grafts, and other adjuncts are not necessary to stabilize the repositioned second molar. All suggestions are acceptable for preventing mobility of the uprighted tooth, leading to optimal healing of periodontal tissues.

Timing is important, especially when the clinician uprights the lower second molar. Some authors did not mention the ideal ages when to upright an ILM2 but did elaborate on the optimum root formation stage, which is one-half to two-thirds root formation. 4 5 10 11 35 The recommended age to start the treatment was 11 years old. 4 11 28 35 Incomplete root formation leads to better prognosis and fewer complications, as open apices allow for ease of vascularization of the pulp complex. 36 The suggested age and corresponding root development by different authors for surgical uprighting are presented in Table 3 .

**Table 3:** Advised age and root development for surgical uprighting of second mandibular molars

Author	Age	Root development
Pogrel 35	11.7–17.9 years old	2/3 root completed with open apices
Dessner 10	Not mentioned	2/3 of root formation
Sawicka et al 28	11–14 years old	Incomplete root formation
Kravitz et al 4	11–15 years old	1/2 to 2/3 root formation
McAboy et al 5	Not mentioned	1/3 to 1/2 of the final root length
Boynton and Leiblich 11	11–14 years	2/3 of root formation

While the presentation of the widely used surgical uprighting is safe and effective for second mandibular molars, it is important to note that case selection and proper knowledge of the second molar root development should be practiced thoroughly. 23 28 37 Patient's cooperation and good oral hygiene are requirements for best possible results. The patient should see the orthodontist to proceed with their respective treatment plan after 1 to 2 weeks. 4 A summary of the retrospective studies and case reports are shown in Tables 4 and 5 , respectively.

**Table 4:** Retrospective studies on surgical uprighting of impacted mandibular second molars

Author	Year	Tooth #	Sample size	Mean age	Follow-up periods	Adjacent third molar status	Outcomes
Pogrel 35	1995	37, 47	16 patients	14.1 years	Minimum of 18 months	4 cases of third molars were not removed	Optimum stabilization was achieved in most cases performed with careful bone reduction
Padwa et al 18	2017	37, 47	16 patients	13 ± 1.1 years	Postoperative radiographs obtained 2.4 ± 1.4 years later	Third molars were removed in 50% of the patients	Pulp obliteration, periapical radiolucency, and root resorption were seen in 31.6% of patients
Caminiti et al 14	2020	37, 47	177 patients with 260 mandibular second molars	14.8 years	6 months	86.9% (266) of third molars removed	255 out of 260 second molar teeth were successfully uprighted from 177 patients

**Table 5:** Case reports on surgical uprighting of impacted mandibular second molars

Author	Year	Second molars	Age, female	Follow-up	Adjacent third molar status	Outcomes
Shipper and Thomadakis 32	2003	37, 47	11 years, female	3 months, 6 months, until 3 years	Third molars removed	#37 normal after 6 months #47: the tooth was endodontically treated twice but eventually healed after 3 years
McAboy et al 5	2003	47	14 years old, female	Up to 3 years	Third molar removed	The tooth was vital

### Soft Tissue Management after Surgical Positioning/Uprighting

There is no definite guideline on soft tissue handling after surgical manipulation of ILM2. It has been emphasized that effective soft tissue management warrants the success of the overall treatment outcomes. However, according to Anderson et al, 3 there is a shortage of studies because of time limitations. The clinician can then acquire careful tissue handling that is consistent with other surgical procedures performed in the lower arch.

Preferably, the uprighted tooth should be reduced on the occlusal surface to be slightly out of contact to prevent occlusal and soft tissue trauma. 5 In a technical note by Anderson et al, 3 they recommended flap repositioning and occlusal composite button placement in terms of soft tissue management. The flap repositioning is needed to minimize tissue trauma of the expected buccal flap created after the said modality. This procedure can be accomplished by doing conservative osteotomy from the marginal buccal bone of the repositioned ILM2 to the third molar extraction area. Once adequate bone removal is achieved, the flap will be approximated from the distal of the second molar then sutured to close.

An occlusal composite button is an added accessory which will raise the bite to keep the flap undisturbed, thereby promoting proper healing. The orthodontist can remove the button during their treatment time, 10 to 14 days after surgical manipulation. Indeed, this method will be difficult to achieve when the third molar is preserved. This method was stated to have advantages like preserving the keratinized buccal gingiva, preventing plaque accumulation, and avoiding hyperocclusion. 3

The occurrence of pseudopockets can also be a major problem for the clinician since there is unpredictable soft tissue behavior after second mandibular molar uprighting. One possible technique to prevent periodontal problems is distal wedge surgery which can be performed in the area of the third lower molars to the second mandibular molar if removal of the third molar is indicated. In this procedure, a triangular incision is made with the apex of the incision located in the direction of the retromolar area and the base located in the distal part of the uprighted second molar. 38 This technique is favorable for cases where the third lower molar is indicated to be extracted. Additionally, it facilitates healthy healing around the second lower molar tissues. This method was applied by Leechanavanichpan et al 39 in 2019, where impacted mandibular third molar was surgically removed with distal wedge incision, and it created positive periodontal clinical outcomes. The distal wedge technique is mostly used during odontectomy procedures and can be beneficial in second mandibular molar uprighting, preventing clinical attachment loss and plaque retention to the distal part of the molar.

Another soft tissue management method is covering the operated site with a periodontal dressing of choice that can be placed to splint the tooth for stabilization and avoid buccal tissue trauma. 4 The dressing can also serve an additional purpose of inhibiting the soft tissue from slowly covering the crown of the uprighted tooth. 14 Prevention of this adverse event is important as its removal can be tedious. In addition, problems such as inflammation of the gingiva and caries formation in the second molar are possible complications of this condition.

Irrigation of the exposed area is also advised postoperatively, using a syringe to prevent food debris accumulation on the area, making the site infection-free. 20 Any other adjuncts for proper care of surgical sites are strongly recommended so that the tooth will not be indicated for subsequent extraction.

### Uprighting with Miniplate and Miniscrew Application

Over the last decade, there have been increases in the number of case studies and reviews regarding miniplates and miniscrews in uprighting ILM2. 40 These devices have been chosen for molar uprighting procedures with orthodontic appliances as they offer stability and anchorage during uprighting. 41 Both anchorage devices were known to have special considerations and shortcomings.

Miniscrews provide optimal stability and the necessary force to move the tooth. They are conservative and inexpensive as well. However, the placement should be done by an experienced clinician, and the precision of placement should be supplemented by three-dimensional imaging for proper installation. 42 In the mandible, special considerations are emphasized since it has a higher amount of cortical bone that correlates with the compromised stability of miniscrews. 43 An interesting finding by Samrit et al 44 revealed that the miniscrew application is predominantly successful in the maxilla than the mandible to a general extent. Nevertheless, many case studies report successful outcomes with uprighting using miniscrews in ILM2.

On the other hand, miniplates are stronger and sturdier than miniscrews, but are expensive. In addition, they require invasive surgery rendering them to be traumatizing. 42

In general, both miniscrews and miniplates are efficient in retraction and distalization on both dental arches. 41 However, there is less concern when it comes to placement with miniplates due to the fact that there is less risk of damaging vital structures. 45

There are two types of miniscrew anchorage: direct anchorage, which involves mounting the miniscrew in the retromolar region; and indirect anchorage, in which placement is situated between the roots of the teeth. 15 Of the two types, the direct anchorage was mentioned to be more favorable for immediate orthodontic loading. 46 An example of direct anchorage is from a 2020 case study by Altieri et al, 16 who used a miniscrew anchorage drilling in the retromolar area along with orthodontic assistance to upright bilateral ILM2. Similarly, Giancotti et al 47 in 2004 placed a 7-mm-long miniscrew in the retromolar triangle to upright a left ILM2 in the same day, with 50 g of force applied by the elastics. Furthermore, from the same author, a 150 g was exerted for a right ILM2 after miniscrew installation on the same area as well. 48 Hence, the force of 50 to 150 g can then be applied on ILM2 uprighting, with cautious manipulation.

In indirect anchorage, an example would be from a case series by Lee et al, 15 where they placed the miniscrew in the buccal cortical bone between the first mandibular molar and second premolars. They used an open coiled spring to maximize the distalizing force to fully upright the ILM2 in its rightful position. Three of the cases presented were adolescents aged 12, 13, and 16 years old, respectively. All of the cases reported success in uprighting via indirect anchorage with miniscrews, even in the case of a 13-year-old patient who did not undergo lower third molar removal. Therefore, miniscrews can be used even if third molar removal is not warranted.

Two miniscrews with slots placed carefully on the buccal alveolus between mandibular first molar and second premolar, and between second premolar and first premolar, can also be installed to increase retention, making the whole setup impervious to orthodontic forces. 49 Compared with the case report by Lee et al, correction with added miniscrews took almost double the time as compared with one miniscrew with open coil springs. The cause of the prolonged time was possibly because some patients in this case series had dislodged miniscrews before the ILM2 got uprighted. Orthodontic biomechanics needed in the use of miniscrews, however, is beyond the scope of this review.

Complex uprighting with miniscrews is also an option, as with Celebi and colleagues' 50 approach on uprighting. The miniscrew was installed in the upper posterior alveolus area for maximum traction from the maxillary arch. Regardless of the location of the miniscrew, uprighting time is the same as the miniscrews placed in the mandibular arch.

Similarly, with miniscrews, miniplates should also be mounted by a clinician with proficient knowledge of the mandibular anatomy as proper flap reflection is required. 51 It is usually performed by oral surgeons. Regardless of the invasiveness of the procedure, the chance of iatrogenic nerve impairment is less concerning. 52 As it stands, there are no known fractures involving miniplates and their subsequent screws as they are known to be very rigid. 42

Most miniplates in the mandible for molar uprighting are placed in the retromolar region, and they can be used for disimpacting deep ILM2. An instance is a clinical report by Tseng et al 52 that utilized a four-hole L-shaped miniplate drilled by miniscrews in the buccal cortex near the retromolar area as an anchorage for orthodontic ILM2 uprighting. The case was corrected in 8 months. Miyahira et al 53 also had a case with a 12-year-old patient in which they used an L-shaped miniplate positioned distal to the ILM2. With the help of optimum orthodontic forces, an ILM2 was nearly uprighted for 3 months. This technique can be considered invasive, but in spite of the trauma it entails, outcomes have been reported to be acceptable.

In the studies mentioned, it is clear that miniscrews have been mostly utilized in ILM2 uprighting cases. Miniplates, however, with their limited studies, should not be concluded lightly. Sherwood et al 51 claimed that miniplates are more stable than other anchorage devices. In contrast, several miniplates in the mandible in a study by Choi et al 54 had failed, with a 7% failure rate. Thus, more studies are needed in this type of modality in ILM2 to determine the exact success rate of this procedure.

Case reports used to upright ILM2 with miniscrews and miniplates have shown that there is correction regardless of the age and degree of root development, as some of the patients already have fully developed second mandibular molar roots. The details are presented in Table 6 .

**Table 6:** Summary of case reports utilizing miniscrews and miniplates for impacted second mandibular molar uprighting

Author	Year	Anchorage device	Tooth number	Age, gender	Time uprighted	Adjacent third molar status
Giancotti et al 47	2004	Miniscrew	37	27 years, male	8 months	Third molar removed before uprighting
Lee et al 15	2007	Miniscrew	47 37 47	12 years, female 13 years, female 16 years, male	5 months 5 months 2 months	Cases 1 and 3 opted for third molar removal, while case 2 uprighting accomplished with adjacent third molar
Nęcka et al 46	2010	Miniscrew	47	15 years, male	6 months	Third molars removed before uprighting
Celebi et al 50	2011	Miniscrew	37	15 years old, male	8 months	Third molar not removed
Mah et al 49	2015	Miniscrews (2)	37, 47 47 37, 47	11 years, female 13 years, female 13 years, male	9 months 13 months 12 months	Agenesis Agenesis Third molar extracted before uprighting
Altieri et al 16	2020	Miniscrew	37, 47	12 years, female	3 months	Third molar extracted before uprighting
Lorente et al 21	2021	Miniscrew	47	13 years, male	4 months	Agenesis
Tseng et al 52	2008	Miniplate	37	19 years, female	8 months	Third molar extracted before uprighting
Miyahira et al 53	2008	Miniplate	#47	12 years, male	3 months	Third molar extracted before uprighting

As per Giancotti et al, 48 wound healing of the surgical site of miniscrew placement takes 10 to14 days. Miniplate healing, on the other hand, is assumed to be longer because of the expected swelling within 7 days post placement and within 7 days after removal. 55 Flapless miniscrew placements have higher success rates with reduced pain and discomfort than those performed with full-thickness flap surgery. In the case of miniplate installation, postoperative pain is experienced with or without flap operation. 56 Patients were also known to be comfortable in the placement and removal of miniscrews, but in the case of miniplates are believed to be otherwise. 47 57 Nevertheless, best oral hygiene practices are advised since these are plaque-retentive fixtures. Patient cooperation is also key to avoid dislodgement and subsequent swallowing of the small components.

### Extraction of the Second Mandibular Molar

The etiologic factors of ILM2 according to Kenrad et al 23 are secondary retention, decreased space, and anomalous inclination. Gooris et al 58 proposed the removal of these ILM2 as there is anticipation that the third molar will eventually erupt toward the extracted second molar's position. Interestingly, Orton-Gibbs et al asserted that third molars, even with severe angulation, can replace these ILM2 effectively after extraction. There is an immense risk as the space of the extracted site does not ensure the eruption of the third molar. 59 60 Known cases of successful replacement were seen in the maxillary arch. 61 However, in the mandibular arch, failed replacements were observed. 62

Clinically, the option to extract the ILM2 is advised if uprighting/repositioning has been deemed impossible because of its detrimental position in the mandibular arch. 63 While radiographically, there are factors to be considered to utilize extraction for effective replacements: proximity of the tooth follicle to the ILM2 roots, the minimal amount of tipping, and crown completion of the third mandibular molar bud. 58

Another contributing indicator for ILM2 extraction is the patient's age, wherein the more advanced the age, the possibility of root closure is anticipated. 63 Despite the difficulties mentioned earlier, mandibular third molar teeth can still erupt in the position of the second molars, but not without deficiencies. A retrospective study by De-la-Rosa-Gay et al 62 resulted only in 66.2% of third mandibular molar eruption to its correct position when the ILM2 was extracted. The failure was accounted for by the later Nolla's stages of development, which is the root closure and completion. This finding is crucial along with Gooris and colleagues' 58 study, in which the optimal time of ILM2 extraction depends on the adjacent third molar's root development. Such time should be from the crown completion to two-thirds root development. The third molars that failed to erupt successfully either had a severe mesial tilt or the proximal contact was not achieved, thereby requiring more orthodontic correction. 62

Correct eruption of third mandibular molars has been mentioned as unpredictable and inconsistent. 5 In terms of arch length, Richardson and Richardson 64 proposed that one can opt for second mandibular molar extraction if the arch length is deficient, with the certainty that the third molar would be impacted and the proper eruption is assumed. However, Magnusson and Kjellberg 26 concluded the opposite for this condition, as they advised that the patient should be cautioned that the final position of the third molars in both arches may be unfavorable, leading to more orthodontic problems such as crossbites. Among all the surgical treatment modalities that transpired in their retrospective study, the second molar extraction with emphasis on the mandibular second molars received the most unsatisfactory results. To address these problems, an additional study by De-la-rosa-Gay et al 65 in 2010 developed a predictive model to estimate the degree of tilting of the eruption of the third molars in both upper and lower teeth by using panoramic radiographs, and by calculating angles and coefficients. The predictive models were successful after several years, except for some mandibular molars mentioned in the study. Although the mandibular results in the study were not as successful as the maxillary results, this can still be an added prognostic value for treatment planning that will involve second molar extractions.

Inconsistencies in some cases are inevitable, so with thorough assessment and treatment planning, eventual ILM2 extractions can be an option. The case Boffano et al presented was extraction by sectioning. In the said case, the ILM2 was deeply impacted, with the third molar situated above it and arranged parallel to each other in a horizontal position. Weighing the risks and benefits is valuable in these kinds of procedures. So, in this particular case, both ILM2s are better to be extracted because of the tediousness of the uprighting procedure and the proximity of the inferior alveolar nerve to the ILM2. Removal of ILM2 was also a preference for Mariano et al 66 since the impacted tooth was very deep with distoangular orientation. Both cases have been synthesized to consider crucial factors such as the patient's age, the thickness of the underlying bone in the area, and the location and position of the ILM2. Details can be seen in Table 7 .

**Table 7:** Case reports of extracted impacted second molars

Author	Year	Study Design	Second molar	Age, gender	Outcomes	Follow-up	Third molar status	Remarks
Mariano et al 66	2006	Case report	#47	26 years old, female	No pain, no paresthesia	1 year: bone healing	Retained	Difficult extraction with sectioning
Boffano et al 63	2010	Case report	#37	19 years old, female	No pain, no paresthesia	6 months: bone healing observed	Retained	Difficult extraction with sectioning

Proper case selection for the ILM2 extraction procedure is essential as most of the outcomes presented were unpredictable. The clinician must also be careful as one disadvantage is possible supraeruption of the opposing tooth which occurs if the tooth is left without contact from the antagonist for prolonged periods, leading to further challenging orthodontic correction. 67 To a large extent, the length of time for correction, the proximity to the inferior alveolar canal, the morphological anomalies of the ILM2, and the overall health of the patient have to be considered. More studies are needed, with suggested emphasis on position and classification of the ILM2 and third molars, and their corresponding management, to assist the clinician in the clinical setting if such a need arises.

### Autotransplantation

As early as the 1950s, autotransplantations or transplantations have been proposed as a replacement modality for extracted teeth. It is broadly known as relocating the tooth from its alveolar socket to another area in the same person. 68 Choosing this treatment option is viable when surgical exposure and orthodontic traction have been unsuccessful. 69 Autotransplantations are often suggested to have a good prognosis as they preserve function, mastication, and proprioception, particularly in young patients. 70 It is a feasible option since dental implants are contraindicated for growing patients due to their maturing alveolar arches. 71 72 Furthermore, orthodontic treatment is more or less unnecessary in this type of modality. 73

Currently, there are no specific guidelines when it comes to the transplantation of teeth. 74 75 Many case reports have shown that it is especially beneficial to second mandibular molar space rehabilitation, due to the general claim that transplantations reduce alveolar resorptions. 76 Moreover, it was attested that this method is less traumatic than performing odontectomy. 77 It is also important that when doing this technique, the donor and recipient teeth should be morphologically analogous to each other to minimize extraoral time, thus maximizing positive results. 13

Case selection for third molar transplantation to the ILM2 site is of utmost importance. Similar to uprighting surgical procedures of the ILM2, autotransplantations of the adjacent third molars were believed to require a certain root development stage for successful outcomes. Radiographic measurements of the third molar root length had been used before, disputing that 2 to 3 mm or 3 to 5mm are the optimal lengths for a good transplantation prognosis. 78 Similar to root development standards in surgical uprighting of ILM2, one-half to three-fourths of root development of the third molar have also been reinforced as an acceptable guideline. 13 26 76 79 80 However, compelling evidence also demonstrated a high success rate with mature third molars with complete root formation. 81 82 83 84 The actual success rates were established on mature teeth because of atraumatic and swift transplantation methods, sufficient alveolar bone dimensions, and aseptic protocols utilized. Despite the success rates, autotransplantations still entail immense risk like periodontal and pulpal complications after the procedure, specifically for mature teeth. 4 5

To date, there are only case reports and retrospective studies that showcased transplantation of third molars to their adjacent second molar extraction sites. Most of them were about extracted second molars that were either decayed or with pulpal pathosis and were not extracted due to impaction or uneruption. 72 79 81 84 85 86 Regardless, three case reports matched our criteria. The first report is one of Clokie and team's 77 case series, where impacted lower right third and second molars were evaluated.

The decision to extract the ILM2 was reinforced since orthodontic correction cannot be performed. To rehabilitate the space, they transplanted the impacted lower third molars tooth adjacent to the ILM2 since it has two-thirds root development. Lai, 87 on the other hand, reported a case where he extracted a left ILM2 with a residual follicle left in the socket. He transplanted the adjacent unerupted third molar with one-fourth root development. Both cases yielded positive results as both teeth proceeded with their root development, and maintained their pulp vitality and function.

Ahmed Asif et al 88 reported a transplantation case with bilateral impacted third and second mandibular molars with completely formed roots. The ILM2s were sectioned and replaced with the atraumatically removed third molars under general anesthesia. As expected, the autotransplanted teeth were endodontically treated after splinting, which was related to the previous statement about complications of transplanting mature teeth. The outcome explains that caution should be prompted in such clinical situations, no matter how short the extraoral time and how atraumatic the procedure was performed. The details of these cases are shown in Table 8 .

**Table 8:** Case reports of autotransplantation involving impacted mandibular second molars

Author	Year	Age/gender	Recipient site	Donor tooth	Root development of the donor tooth	Status of transplanted tooth	Outcomes	Remarks
Clokie et al 77	2001	17-year-old, female	#47	#48	2/3 root formation	Not mentioned	Successful	Both teeth were impacted
Lai 87	2009	14-year-old, male	#37	#38	1/4 root formation	3 months of progressive root formation of transplanted tooth	Tooth in occlusal level in 7 months with new bone formation observed	Recipient socket was intended to still have residual follicle from an extracted tooth
Ahmed Asif 88	2017	24-year-old, female	#37, #47	#38, #48	Complete root formation	Endodontic treatment performed after splinting	Bone healing 1 year after completion of treatment	Second impacted molars removed by sectioning

Three existing case reports in managing ILM2 are not sufficient to draw a significant conclusion. However, we can derive from long-term case studies and retrospective studies that immature third mandibular molars transplanted to second molar recipient sites were more successful in growing patients with good oral hygiene. 83 86 89

Overall, proper case selection and patient's cooperation are essential to autotransplantations as the outcomes are variable and unpredictable. Many contributing factors can affect the treatment as there is no conclusive evidence and no prevailing guidelines to date. 75 90 Undoubtedly, the benefits outweigh the risks of performing the transplantation. Constantly reminding the patient about maintaining oral hygiene, attending yearly recalls, and checking the status of the implanted tooth are minor inconveniences but will tremendously help in the success of the treatment.

### Third Molar Extractions

Terry and Hegtvedt 12 in 1993 stated that mandibular third molars are one of the contributing factors of malpositioned second mandibular molars; therefore, its removal is required. Pogrel 35 favored the idea as the ease of uprighting can be attained without the presence of the third molar beside the ILM2. He did not extract the third molars in his cases because their proximity does not affect the ILM2 and simply because the third molar buds are invisible radiographically. According to Going and Reyes-Lois, 20 the presence of a third molar does not affect the results promised in the surgical uprighting procedure. Furthermore, Johnson and Taylor 91 provided indirect testimony for this claim. The knowledge of the mandibular third molar eruption pattern in this matter comes to play. However, it is observed that either option is not a factor in expediting the treatment time since both options from our case reports achieved uprighting of the tooth within a maximum of 13 months.

Nonetheless, several authors have articulated that mandibular third molar extraction cases are unnecessary for surgical uprighting procedures. 18 92 93 One reason for unnecessary removal is the presence of a spatial relationship between the third mandibular molars and the second mandibular molars that can aid in the latter's uprighting. 94 With all things considered, it is up to the clinician whether the extraction of adjacent third molar can simplify the treatment.

## Future Directions

Since only limited studies were found in every scope of surgical uprighting of ILM2, documentation is encouraged with detailed specifics, including the classification and position of the adjacent third molar, root development, and proximity to lingual nerve and inferior alveolar nerve, and other related aspects of ILM2. These details will be helpful in further understanding the etiology of such occurrences. Clearer methodologies can help draw definitive conclusions, consequently setting a standard guideline for managing these anomalies properly. Variation of treatment on bilateral cases is especially helpful, but weighing risk and benefits to such cases is still essential. Specifically, in the case of third lower molar extractions, a bilateral comparison between the retained and extracted third lower molar while uprighting its adjacent second molar on the same patient will be valuable in contributing to the discussion.

Prescribing analgesics is common practice, especially after surgical manipulations involving ILM2. 5 20 In particular, ibuprofen can be given preoperatively or postoperatively for pain management. 16 93 Dexamethasone is a corticosteroid used to prevent postoperative pain, trismus, and edema after impacted third molar surgeries, 66 71 and its effect can be advantageous after ILM2 surgical procedures, specifically with surgical uprighting by luxation, miniplates, sectioning, and autotransplantations. The outcomes can be beneficial, since postoperative complications are anticipated. The possibility of neuropathic and chronic pain can occur in difficult cases, so chronic pain management protocols are also suggested to be given. 95

The latest trends that can be used efficiently in our particular case of autotransplantation are three-dimensional printed models and CARP (computer-aided rapid prototyping) for creating treatment planning templates. These three-dimensional adjuncts are superior to radiographic imaging as they can approximate the morphology of the third mandibular molar to be placed in the socket. Thus, the predictability of the placement is expected in a short time. 96 97 98 99 100 Simply employing these innovative techniques can add to the diagnostic value and can contribute to the predictability of ILM2.

Bone grafting procedures are also incorporated in mandibular third molar transplantations as a supplementary technique since inadequate buccolingual width and overpreparation of the transplant site may lead to eventual resorption of the alveolar buccal bone, which can yield a poor prognosis. 76 81 82 Grafting materials like xenografts and autogenous tooth–bone graft can be used on areas with deficient bone, as well as collagen membranes. 101 102 It is necessary to compare the long-term results of such to determine which bone graft material is preferred in these cases.

Comparison of wound healing materials is another path to be studied pertaining to the aforementioned studies to lessen the postoperative sequelae expected.

Published cases about autotransplantations regarding replacement of second mandibular molars have focused more on the adjacent third molar as the donor tooth. However, it will be interesting to see outcomes of the transplanted site from an upper molar as a donor tooth or the contralateral mandibular molar. The results can be furthered as one of the treatment options if the clinician is faced with a situation where the adjacent third molar is not available. This can expand the knowledge domain on autotransplantation as an excellent elective procedure as more options are presented.

Advancements in autotransplantation using piezosurgery have also been used in a third molar to be transplanted into a second molar socket. Atraumatic harvesting of the donor tooth to the recipient site using this device leads to unharmed periodontal ligament fibers that can count for less occurrence of complications. The piezosurgery can also be used for noninvasive socket preparation because of its ability to remove unnecessary bone judiciously. 19 79 More studies on utilization of this device on ILM2 and third molars are recommended.

Quality-of-life assessments should also be undertaken as most of these procedures are rather invasive. The miniscrews and miniplates located in the retromolar area seem to be uncomfortable to patients during function. Thus, the quality-of-life assessment studies can help the clinician choose which modality the patient will comply with.

Updated studies on surgical techniques regarding ILM2 are required since advanced trends in the orthodontic and surgical fields are evolving. It is also needed for a better understanding of these occurrences. Lastly, the necessity for long-term follow-up studies is also advised to ensure the effectiveness of every modality discussed.

## Limitations

Few case reports and retrospective studies spanning more than 20 years are available regarding the management of impacted second molars. The authors of each study made assumptions that are all substantial in contributing to management and case selection. The points made are sufficient to guide the decision-making process, but robust standards cannot be made. Although most of the findings are not current, the case reports and retrospective studies are still relevant to techniques applied in the modern setting, reinforcing the effectiveness of the surgical modality.

## Conclusion

This review covered the surgical aspects of uprighting, repositioning, and autotransplantation, with novel approaches regarding ILM2. Various works have already documented well-known success rates and prognoses for surgical exposures, surgical uprighting, and transplantation, especially presented by retrospective studies. In addition, surgical approaches regarding ILM2 are less complicated than orthodontic uprighting techniques because they require lesser tools with fewer steps. Hence, these surgical modalities are not time-consuming. All in all, the outcome of surgical intervention depends on case selection, root development of the ILM2, careful surgical manipulation, and adherence to sound biological principles. Patient's compliance, general health, and oral hygiene should also be highlighted. Nevertheless, there is a constant need for other innovative, advanced, and atraumatic surgical approaches for ILM2 to give patients more options to improve compliance and comfort.

**Authors' Contributions**

**Table:** 

Authors' contributions	Name of authors
Conceptualization	D.I.S.
Methodology	D.I.S.
Validation	D.R.
Formal analysis	N.W.
Investigation	N.W.
Resources	D.R.
Writing—original draft preparation	D.I.S.
Writing—review and editing	D.R.
Visualization	N.W.
Supervision	N.W.
Project administration	D.R., N.W.
Final approval	N.W.
Agreed to be accountable	N.W.
